# Crystal structure and Hirshfeld surface analysis of ethyl 2-[9-(2-hy­droxy­phen­yl)-3,3,6,6-tetra­methyl-1,8-dioxo-2,3,4,4a,5,6,7,8a,9,9a,10,10a-dodeca­hydro­acridin-10-yl]acetate

**DOI:** 10.1107/S2056989021001341

**Published:** 2021-02-12

**Authors:** Omyma A. A. Abd Allah, Manpreet Kaur, Mehmet Akkurt, Shaaban K. Mohamed, Jerry P. Jasinski, Sahar M. I. Elgarhy

**Affiliations:** a Chemistry Department, Faculty of Science, Sohag University, 82524 Sohag, Egypt; b Department of Chemistry, Keene State College, 229 Main Street, Keene, NH 03435-2001, USA; c Department of Physics, Faculty of Sciences, Erciyes University, 38039 Kayseri, Turkey; d Chemistry and Environmental Division, Manchester Metropolitan University, Manchester M1 5GD, England; e Chemistry Department, Faculty of Science, Minia University, 61519 El-Minia, Egypt; f Faculty of Science, Department of Bio Chemistry, Beni Suef University, Beni Suef, Egypt

**Keywords:** crystal structure, 3,3,6,6-tetra­methyl­tetra­hydro­acridine-1,8-dione, C—H⋯O hydrogen bonds, O—H⋯O hydrogen bonds, acridines

## Abstract

In the title compound, C_27_H_33_NO_5_, a 3,3,6,6-tetra­methyl­tetra­hydro­acridine-1,8-dione ring system carries an ethyl acetate substituent on the acridine N atom and an *o*-hy­droxy­phenyl ring on the central methine C atom of the di­hydro­pyridine ring. The benzene ring is inclined to the acridine ring system at an angle of 80.45 (7)° and this conformation is stabilized by an intra­molecular O—H⋯O hydrogen bond between the hy­droxy substituent on the benzene ring and one of the carbonyl groups of the acridinedione unit. The ester C=O oxygen atom is disordered over major and minor orientations in a 0.777 (9):0.223 (9) ratio and the terminal –CH_3_ unit of the ethyl side chain is disordered over two sets of sites in a 0.725 (5): 0.275 (5) ratio. In the crystal, C—H⋯O hydrogen bonds combine to link the mol­ecules into a three-dimensional network. van der Waals H⋯H contacts contribute the most to the Hirshfeld surface (66.9%) followed by O⋯H/H⋯O (22.1%) contacts associated with weak hydrogen bonds.

## Abstract 2

The mol­ecular conformation is stabilized by an intra­molecular O—H⋯O hydrogen bond between the hy­droxy substituent on the benzene ring and one of the carbonyl groups of the acridinedione unit.

## Chemical context   

Acridine derivatives occur in a number of compounds of importance in medicinal chemistry such as bucricaine, which used for surface anesthesia of the eye and given by injection for infiltration anesthesia, peripheral nerve block and spinal anesthesia (Ramesh *et al.*, 2012 ▸). Quinacrine, also known as mepacrine, is used as a gametocytocide and acts as an anti­malarial agent (Valdés, 2011 ▸). Proflavin is also found to be active as a bacteriostatic agent (Patel *et al.*, 2010 ▸) and nitracrine is as anti­cancer agent (Cholewinski *et al.*, 2011 ▸). Acriflavin is used as an anti­septic for skin and mucous membranes (Ramesh *et al.*, 2012 ▸). As part of our studies in this area, we report herein the synthesis and crystal structure of the title compound, C_27_H_33_NO_5_.

## Structural commentary   

**Figure 1:**
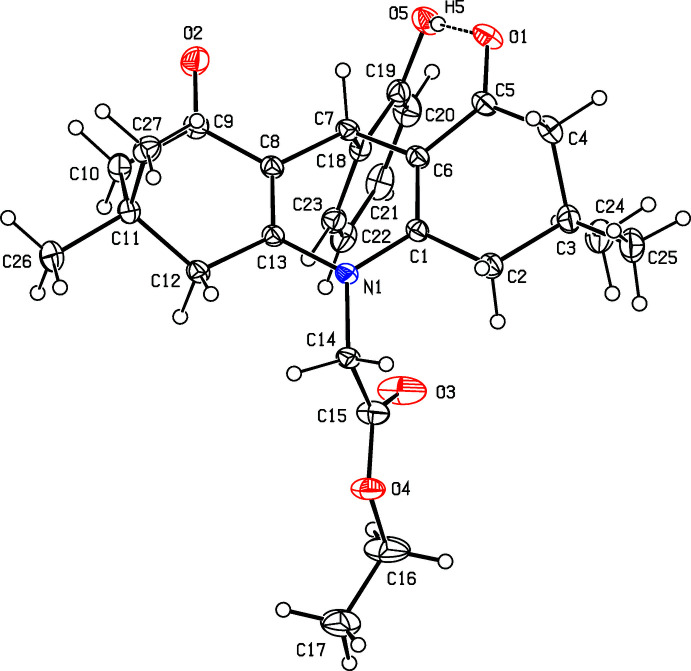
The title mol­ecule with displacement ellipsoids drawn at the 30% probability level. Only the major disorder components for O3 and C17 are shown.

As shown in Fig.1, the 3,3,6,6-tetra­methyl­tetra­hydro­acridine-1,8-dione ring system carries an ethyl acetate substituent on the acridine N1 atom and an *o*-hy­droxy­phenyl ring on the central methine C7 atom of the C1/C6–C8/C13/N1 di­hydro­pyridine ring. The acridinedione ring system deviates significantly from planarity with an r.m.s. deviation of 0.404 Å for the thirteen C atoms and one N atom of the acridine unit. The benzene ring is inclined to the acridine ring system at a dihedral angle of 80.45 (7)°.
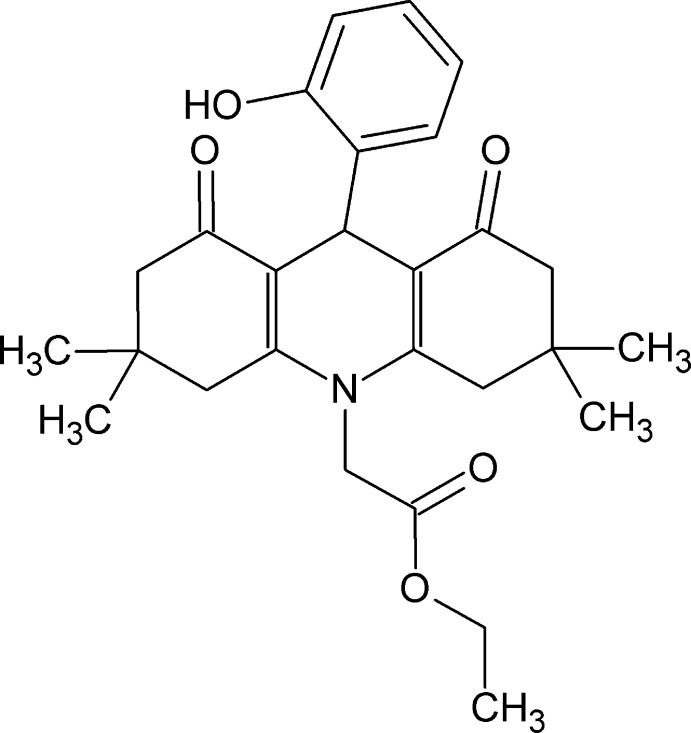


The outer C1–C6 and C8–C13 cyclo­hexenone rings both adopt flattened chair conformations with the C4 and C11 atoms displaced in the same direction, by 0.308 (2) and 0.338 (2) Å, respectively, from the best-fit planes through the remaining five C atoms. In contrast, the central C13/N1/C1/C6–C8 ring can best be described as a flattened boat with N1 and C7 displaced by 0.146 (1) and 0.191 (14) Å, respectively, from the remaining four C atoms. The bond lengths and angles in the title mol­ecule agree reasonably well with those found in closely related mol­ecules (Abdelhamid *et al.*, 2011 ▸, 2014 ▸; Khalilov *et al.*, 2011 ▸).

The mol­ecular conformation of the title compound is stabilized by an intra­molecular O5—H5⋯O1 hydrogen bond between the hy­droxy substituent on the benzene ring and one of the carbonyl groups of the acridinedione unit (Table 1 ▸; Fig. 1 ▸). Atom O3 is disordered over major and minor orientations in a 0.777 (9):0.223 (9) ratio and the terminal C17 methyl group is disordered over two sets of sites in a 0.725 (5):0.275 (5) ratio.

## Supra­molecular features   

**Figure 2:**
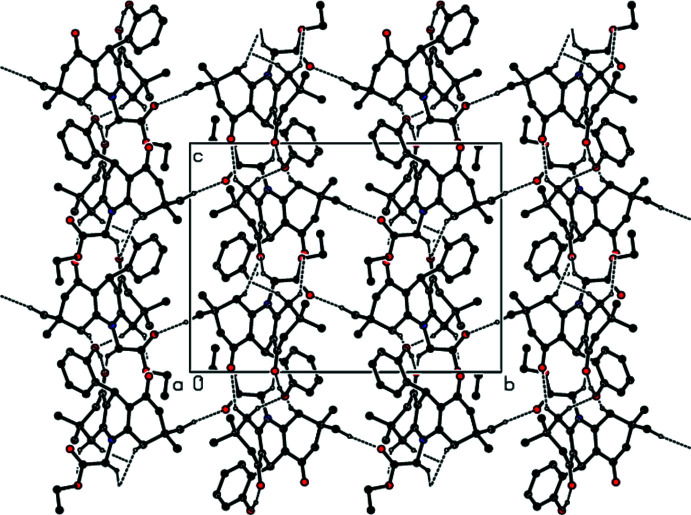
The mol­ecular packing, viewed down the *a-*axis direction, showing hydrogen bonds as dashed lines.

In the crystal, a number of C—H⋯O hydrogen bonds link the mol­ecules into a three-dimensional network (Table 1 ▸; Fig. 2 ▸); all the oxygen atoms in the mol­ecule except O4 accept at least one of these bonds.

## Hirshfeld surface analysis   

The *CrystalExplorer* software (Wolff *et al.*, 2012 ▸) was used to produce the *d*_norm_-mapped Hirshfeld surfaces and the electrostatic potential for the title compound. The contact distances, *d*_i_ and *d*_e_, from the Hirshfeld surface to the nearest atom, inside and outside, respectively, enable the analysis of the inter­molecular inter­actions through the mapping of *d*_norm_. An illustration of the inter-mol­ecular contacts in the crystal is given by two-dimensional fingerprint plots.

**Figure 3:**
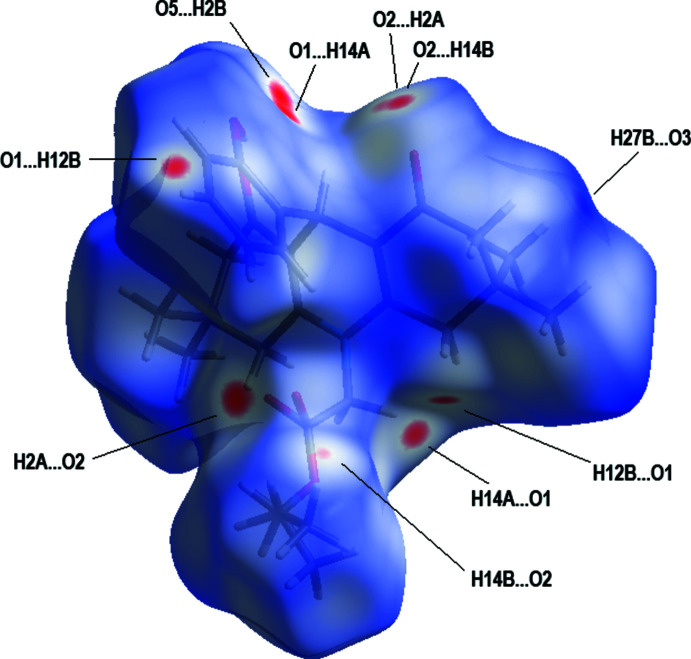
A view of the three-dimensional Hirshfeld surface for the title compound, plotted over *d*_norm_ in the range −0.14 to 1.68 a.u.

The bright-red spots on the Hirshfeld surface mapped over *d*_norm_ (Fig. 3 ▸), with labels H27*B*, H12*B*, H14*A*, H14*B*, H2*A* and H2*B* on the surface represent donors for potential C—H⋯O hydrogen bonds (see Table 1 ▸); the corresponding acceptors on the surface appear as bright-red spots at atoms O1, O2 and O5. Short H⋯H contacts are given in Table 2 ▸.

**Figure 4:**
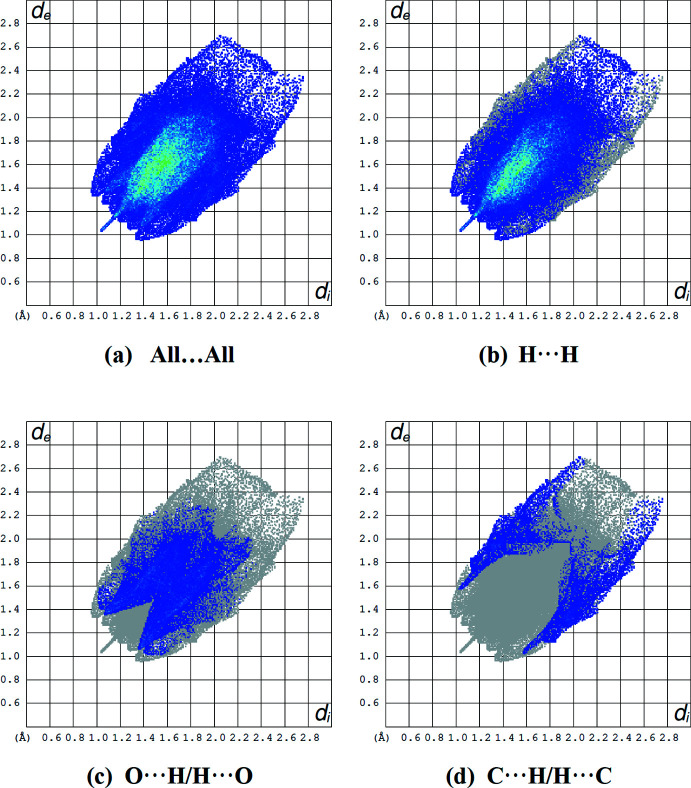
A view of the two-dimensional fingerprint plots for the title compound, showing (*a*) all inter­actions, and delineated into (*b*) H⋯H, (*c*) O⋯H/H⋯O and (*d*) C⋯H/H⋯C inter­actions. The *d*_i_ and *d*_e_ values are the closest inter­nal and external distances (in Å) from given points on the Hirshfeld surface.

The overall two-dimensional fingerprint plot is illustrated in Fig. 4 ▸ *a*, and those delineated into H⋯H, O⋯H/H⋯O and C⋯H/H⋯C in Fig. 4 ▸ *b*–*d*, respectively. The greatest contribution to the overall Hirshfeld surface, *i.e*. 66.9%, is due to H⋯H contacts (Fig. 4 ▸ *b*). The relative contributions of the other inter­actions in descending order are: O⋯H/H⋯O (22.1%), C⋯H/H⋯C (9.2%), O⋯O (1.3%), N⋯H/H⋯N (0.2%) and N⋯C/C⋯N (0.2%). This illustrates that the C—H⋯O inter­actions contribute significantly to the crystal packing.

## Database survey   

Compounds similar to the title compound with a octa­hydro­acridin moiety are [9-(2-hy­droxy­phen­yl)-1,8-dioxo-2,3,4,5,6,7,8,9-octa­hydro­acridin-10(1*H*)-yl]acetic acid [Cam­bridge Structural Database (Groom *et al.*, 2016 ▸)] refcode DABSAD; Akkurt *et al.*, 2015 ▸), ethyl [9-(5-bromo-2-hy­droxyphen­yl)-3,3,6,6-tetra­methyl-1,8-dioxo-2,3,4,5,6,7,8,9-octa­hydro­acridin-10(1*H*)-yl]acetate (VANBUK; Mohamed *et al.*, 2017 ▸), 9-(3-bromo-5-chloro-2-hy­droxy­phen­yl)-10-(2-hy­droxy­eth­yl)-3,6-diphenyl-3,4,6,7,9,10-hexa­hydro­acridine-1,8(2*H*,5*H*)-dione (SILBIB; Abdelhamid *et al.*, 2018 ▸) and 10-benzyl-9-(3,4-di­meth­oxy­phen­yl)-3,3,6,6-tetra­methyl-3,4,6,7,9,10-hexa­hydro­acridine-1,8(2*H*,5*H*)-dione (PUSJEU; Sureshbabu *et al.*, 2015 ▸).

The DABSAD compound crystallizes with two mol­ecules in the asymmetric unit. In each mol­ecule, the central 1,4-di­hydro­pyridine ring adopts a shallow sofa conformation (with the C atom bearing the phenol ring as the flap), whereas the pendant cyclo­hexene rings both have twisted-boat conform­ations. Each mol­ecule features an intra­molecular O—H⋯O hydrogen bond, which closes an *S*(8) ring. In the crystal, the mol­ecules are linked by O—H⋯O, C—H⋯O and C—H⋯π inter­actions, forming a three-dimensional network. In VANBUK, the central 1,4-di­hydro­pyridine ring adopts a shallow sofa conformation (with the C atom bearing the bromo­phenol ring as the flap), whereas the pendant cyclo­hexene rings both have twisted-boat conformations. The mol­ecule features an intra­molecular O—H⋯O hydrogen bond, which closes an *S*(8) ring. In the crystal, mol­ecules are linked by C—H⋯O inter­actions, forming *C*(12) chains propagating along the *c*-axis direction. In the crystal of SILBIB, O—H⋯O, C—H⋯O and C—H⋯π(ring) hydrogen bonds combine with an Br—O and unusual C—Br⋯π(ring) halogen bonds to generate a three dimensional network with mol­ecules stacked along the *a*-axis direction. In the acridinedione moiety of PUSJEU, the central di­hydro­pyridine ring adopts a flattened-boat conformation, with the N atom and the methine C atom displaced from the mean plane of the other four atoms by 0.0513 (14) and 0.1828 (18) Å, respectively. The two cyclo­hexenone rings adopt envelope conformations, with the tetra­subsituted C atoms as the flap atoms. In the crystal, mol­ecules are linked *via* a pair of C—H⋯O hydrogen bonds, forming inversion dimers, which are, in turn, linked by C—H⋯O hydrogen bonds, forming slabs lying parallel to (001).

## Synthesis and crystallization   

To a mixture of dimedone (1.12 g, 0.008 mol), ethyl glycinate hydro­chloride (0.56 g, 0.004 mol) and salicaldehyde (0.43 ml, 0.004 mol) in ethanol (20 ml), triethyl amine (1.12 ml, 0.008 mol) was added. The reaction mixture was heated under reflux for 5 h at 353–358 K then left to cool. The separated solid was filtered off, dried and recrystallized from ethanol solution as yellow plates of the title compound, yield 68%, m.p. 497 K.

## Refinement   

Crystal data, data collection and structure refinement details are summarized in Table 3 ▸. All H atoms were placed in idealized locations and and refined using a riding model with C—H = 0.9–1.00 Å *U*_iso_(H) = 1.2*U*_eq_ (C) and O—H = 0.84 Å, *U*_iso_(H) = 1.5*U*_eq_ (O). Atom O3 of the oxo group and terminal methyl group (C17) of the ethyl acetate substituent are disordered over two sites in 0.777 (9):0.223 (9) (for O3 and O3*A*) and 0.725 (5):0.275 (5) (for C17 and C17*A*) ratios, respectively.

## Supplementary Material

Crystal structure: contains datablock(s) global, I. DOI: 10.1107/S2056989021001341/hb7967sup1.cif

Structure factors: contains datablock(s) I. DOI: 10.1107/S2056989021001341/hb7967Isup2.hkl

Supporting information file. DOI: 10.1107/S2056989021001341/hb7967Isup3.cml

CCDC reference: 2061379

Additional supporting information:  crystallographic information; 3D view; checkCIF report

## Figures and Tables

**Table 1:** Hydrogen-bond geometry (Å, °)

*D*—H⋯*A*	*D*—H	H⋯*A*	*D*⋯*A*	*D*—H⋯*A*
O5—H5⋯O1	0.84	1.81	2.6319 (17)	166
C2—H2*A*⋯O2^i^	0.99	2.52	3.1663 (19)	123
C2—H2*B*⋯O5^i^	0.99	2.53	3.457 (2)	157
C12—H12*B*⋯O1^ii^	0.99	2.52	3.2708 (17)	133
C14—H14*A*⋯O1^ii^	0.99	2.53	3.3299 (18)	138
C14—H14*B*⋯O2^i^	0.99	2.66	3.473 (2)	140
C27—H27*B*⋯O3^iii^	0.98	2.51	3.452 (3)	161

Symmetry codes: (i) x+{\script{1\over 2}}, -y+{\script{1\over 2}}, z+{\script{1\over 2}}; (ii) x-{\script{1\over 2}}, -y+{\script{1\over 2}}, z+{\script{1\over 2}}; (iii) -x+{\script{1\over 2}}, y+{\script{1\over 2}}, -z+{\script{3\over 2}}.

**Table 2:** Short H⋯H inter­atomic contacts (Å) in the title compound

Contact	Distance	Symmetry operation
H21⋯H27*A*	2.26	−{1\over 2} + *x*, {1\over 2} − *y*, −{1\over 2} + *z*
H22⋯H27*A*	2.46	{1\over 2} − *x*, −{1\over 2} + *y*, {3\over 2} − *z*
H22⋯H4*B*	2.43	−1 + *x*, *y*, *z*

**Table 3:** Experimental details

Crystal data	
Chemical formula	C_27_H_33_NO_5_
*M*_r_	451.54
Crystal system, space group	Monoclinic, *P*2_1_/*n*
Temperature (K)	173
*a*, *b*, *c* (Å)	9.5289 (2), 18.6653 (5), 13.8046 (3)
β (°)	96.410 (2)
*V* (Å^3^)	2439.93 (10)
*Z*	4
Radiation type	Cu *K*α
μ (mm^−1^)	0.68
Crystal size (mm)	0.48 × 0.22 × 0.08

Data collection	
Diffractometer	Rigaku Oxford Diffraction EOS
Absorption correction	Multi-scan (*CrysAlis PRO*; Rigaku OD, 2015 ▸)
*T*_min_, *T*_max_	0.861, 1.000
No. of measured, independent and observed [*I* > 2σ(*I*)] reflections	9527, 4648, 3949
*R*_int_	0.029
(sin θ/λ)_max_ (Å^−1^)	0.614

Refinement	
*R*[*F* ^2^ > 2σ(*F* ^2^)], *wR*(*F* ^2^), *S*	0.045, 0.126, 1.04
No. of reflections	4648
No. of parameters	313
H-atom treatment	H-atom parameters constrained
Δρ_max_, Δρ_min_ (e Å^−3^)	0.27, −0.23

Computer programs: *CrysAlis PRO* (Rigaku OD, 2015 ▸), *SHELXT* (Sheldrick, 2015 ▸b), *SHELXL* (Sheldrick, 2015 ▸a) and *OLEX2* (Dolomanov *et al.*, 2009 ▸).

## supplementary crystallographic information

### Crystal data

**Table:**

C_27_H_33_NO_5_	*F*(000) = 968
*M**_r_* = 451.54	*D*_x_ = 1.229 Mg m^−^^3^
Monoclinic, *P*2_1_/*n*	Cu *K*α radiation, λ = 1.54184 Å
*a* = 9.5289 (2) Å	Cell parameters from 3784 reflections
*b* = 18.6653 (5) Å	θ = 4.0–71.4°
*c* = 13.8046 (3) Å	µ = 0.68 mm^−^^1^
β = 96.410 (2)°	*T* = 173 K
*V* = 2439.93 (10) Å^3^	Plate, yellow
*Z* = 4	0.48 × 0.22 × 0.08 mm

### Data collection

**Table:**

Rigaku Oxford Diffraction EOS diffractometer	4648 independent reflections
Radiation source: fine-focus sealed X-ray tube, Enhance (Cu) X-ray Source	3949 reflections with *I* > 2σ(*I*)
Graphite monochromator	*R*_int_ = 0.029
Detector resolution: 16.0416 pixels mm^-1^	θ_max_ = 71.2°, θ_min_ = 4.0°
ω scans	*h* = −11→11
Absorption correction: multi-scan (CrysalisPro; Rigaku OD, 2015)	*k* = −22→14
*T*_min_ = 0.861, *T*_max_ = 1.000	*l* = −16→16
9527 measured reflections	

### Refinement

**Table:**

Refinement on *F*^2^	Primary atom site location: dual
Least-squares matrix: full	Hydrogen site location: inferred from neighbouring sites
*R*[*F*^2^ > 2σ(*F*^2^)] = 0.045	H-atom parameters constrained
*wR*(*F*^2^) = 0.126	*w* = 1/[σ^2^(*F*_o_^2^) + (0.0643*P*)^2^ + 0.6477*P*] where *P* = (*F*_o_^2^ + 2*F*_c_^2^)/3
*S* = 1.04	(Δ/σ)_max_ < 0.001
4648 reflections	Δρ_max_ = 0.27 e Å^−^^3^
313 parameters	Δρ_min_ = −0.23 e Å^−^^3^
0 restraints	

### Special details

**Table:**

Geometry. All esds (except the esd in the dihedral angle between two l.s. planes) are estimated using the full covariance matrix. The cell esds are taken into account individually in the estimation of esds in distances, angles and torsion angles; correlations between esds in cell parameters are only used when they are defined by crystal symmetry. An approximate (isotropic) treatment of cell esds is used for estimating esds involving l.s. planes.

### Fractional atomic coordinates and isotropic or equivalent isotropic displacement parameters (Å^2^)

**Table:**

	*x*	*y*	*z*	*U*_iso_*/*U*_eq_	Occ. (<1)
O1	0.65518 (12)	0.22713 (7)	0.50403 (8)	0.0373 (3)	
O2	0.21715 (14)	0.36821 (7)	0.51857 (8)	0.0426 (3)	
O3	0.3445 (5)	0.11473 (19)	0.83632 (16)	0.0636 (11)	0.777 (9)
O3A	0.2813 (17)	0.1388 (6)	0.8394 (7)	0.0636 (11)	0.223 (9)
O4	0.35606 (14)	0.14006 (6)	0.99586 (9)	0.0406 (3)	
O5	0.41985 (13)	0.19489 (7)	0.39430 (8)	0.0389 (3)	
H5	0.4887	0.2122	0.4300	0.058*	
N1	0.44568 (13)	0.24991 (6)	0.79603 (8)	0.0236 (2)	
C1	0.54686 (14)	0.22154 (7)	0.74277 (10)	0.0228 (3)	
C2	0.66871 (16)	0.18305 (8)	0.79873 (11)	0.0292 (3)	
H2A	0.6309	0.1464	0.8407	0.035*	
H2B	0.7234	0.2179	0.8419	0.035*	
C3	0.76892 (16)	0.14645 (9)	0.73461 (12)	0.0328 (3)	
C4	0.79512 (16)	0.19849 (9)	0.65285 (12)	0.0356 (4)	
H4A	0.8424	0.2421	0.6814	0.043*	
H4B	0.8587	0.1758	0.6098	0.043*	
C5	0.65908 (16)	0.21917 (8)	0.59366 (11)	0.0286 (3)	
C6	0.53718 (15)	0.23162 (8)	0.64433 (10)	0.0245 (3)	
C7	0.40195 (15)	0.25806 (8)	0.58758 (10)	0.0244 (3)	
H7	0.4283	0.2880	0.5323	0.029*	
C8	0.32755 (14)	0.30588 (7)	0.65354 (10)	0.0237 (3)	
C9	0.22950 (16)	0.35939 (8)	0.60657 (11)	0.0291 (3)	
C10	0.14223 (16)	0.40152 (9)	0.67124 (12)	0.0323 (3)	
H10A	0.0581	0.3731	0.6833	0.039*	
H10B	0.1091	0.4463	0.6376	0.039*	
C11	0.22671 (16)	0.42010 (8)	0.76873 (11)	0.0280 (3)	
C12	0.27846 (15)	0.34965 (8)	0.81805 (10)	0.0263 (3)	
H12A	0.3461	0.3608	0.8758	0.032*	
H12B	0.1970	0.3245	0.8411	0.032*	
C13	0.34886 (14)	0.30040 (7)	0.75185 (10)	0.0222 (3)	
C14	0.43949 (15)	0.22835 (8)	0.89738 (10)	0.0256 (3)	
H14A	0.3858	0.2646	0.9304	0.031*	
H14B	0.5365	0.2265	0.9315	0.031*	
C15	0.37019 (19)	0.15605 (9)	0.90423 (12)	0.0368 (4)	
C16	0.2856 (3)	0.07304 (12)	1.01388 (17)	0.0641 (7)	
H16A	0.3553	0.0335	1.0218	0.077*	0.725 (5)
H16B	0.2146	0.0615	0.9581	0.077*	0.725 (5)
H16C	0.2872	0.0420	0.9559	0.077*	0.275 (5)
H16D	0.3405	0.0485	1.0693	0.077*	0.275 (5)
C17	0.2157 (4)	0.08124 (19)	1.1040 (3)	0.0665 (9)	0.725 (5)
H17A	0.2875	0.0880	1.1598	0.100*	0.725 (5)
H17B	0.1604	0.0381	1.1141	0.100*	0.725 (5)
H17C	0.1530	0.1230	1.0976	0.100*	0.725 (5)
C17A	0.1551 (11)	0.0790 (5)	1.0331 (8)	0.0665 (9)	0.275 (5)
H17D	0.1499	0.1142	1.0853	0.100*	0.275 (5)
H17E	0.1217	0.0324	1.0541	0.100*	0.275 (5)
H17F	0.0956	0.0947	0.9745	0.100*	0.275 (5)
C18	0.30828 (15)	0.19663 (8)	0.54418 (10)	0.0258 (3)	
C19	0.32270 (16)	0.16932 (9)	0.45105 (11)	0.0307 (3)	
C20	0.23375 (19)	0.11504 (10)	0.41156 (13)	0.0405 (4)	
H20	0.2428	0.0976	0.3479	0.049*	
C21	0.13242 (18)	0.08647 (9)	0.46441 (14)	0.0410 (4)	
H21	0.0723	0.0493	0.4371	0.049*	
C22	0.11833 (17)	0.11164 (9)	0.55659 (13)	0.0356 (4)	
H22	0.0494	0.0916	0.5933	0.043*	
C23	0.20564 (16)	0.16652 (8)	0.59556 (11)	0.0295 (3)	
H23	0.1949	0.1839	0.6590	0.035*	
C24	0.7045 (2)	0.07682 (10)	0.69143 (15)	0.0469 (4)	
H24A	0.6894	0.0436	0.7443	0.070*	
H24B	0.7688	0.0550	0.6493	0.070*	
H24C	0.6139	0.0872	0.6531	0.070*	
C25	0.9082 (2)	0.12973 (11)	0.79789 (14)	0.0468 (4)	
H25A	0.9533	0.1746	0.8214	0.070*	
H25B	0.9713	0.1036	0.7590	0.070*	
H25C	0.8887	0.1003	0.8537	0.070*	
C26	0.13320 (19)	0.45952 (9)	0.83469 (13)	0.0405 (4)	
H26A	0.0495	0.4305	0.8424	0.061*	
H26B	0.1038	0.5057	0.8052	0.061*	
H26C	0.1864	0.4677	0.8987	0.061*	
C27	0.35163 (17)	0.46822 (8)	0.75087 (12)	0.0340 (3)	
H27A	0.4061	0.4799	0.8133	0.051*	
H27B	0.3163	0.5125	0.7187	0.051*	
H27C	0.4123	0.4431	0.7092	0.051*	

### Atomic displacement parameters (Å^2^)

**Table:**

	*U*^11^	*U*^22^	*U*^33^	*U*^12^	*U*^13^	*U*^23^
O1	0.0375 (6)	0.0503 (7)	0.0266 (6)	−0.0016 (5)	0.0144 (4)	−0.0032 (5)
O2	0.0548 (7)	0.0469 (7)	0.0249 (6)	0.0155 (6)	−0.0006 (5)	0.0006 (5)
O3	0.114 (3)	0.0437 (16)	0.0360 (8)	−0.0364 (17)	0.0192 (12)	−0.0122 (10)
O3A	0.114 (3)	0.0437 (16)	0.0360 (8)	−0.0364 (17)	0.0192 (12)	−0.0122 (10)
O4	0.0571 (7)	0.0352 (6)	0.0317 (6)	−0.0098 (5)	0.0158 (5)	0.0055 (5)
O5	0.0448 (6)	0.0485 (7)	0.0245 (5)	0.0025 (5)	0.0080 (5)	−0.0075 (5)
N1	0.0288 (6)	0.0242 (6)	0.0189 (5)	0.0020 (5)	0.0069 (4)	−0.0005 (4)
C1	0.0248 (6)	0.0203 (6)	0.0241 (7)	−0.0020 (5)	0.0064 (5)	−0.0023 (5)
C2	0.0305 (7)	0.0316 (7)	0.0257 (7)	0.0041 (6)	0.0035 (6)	−0.0014 (6)
C3	0.0324 (8)	0.0323 (8)	0.0344 (8)	0.0071 (6)	0.0065 (6)	−0.0027 (6)
C4	0.0290 (8)	0.0417 (9)	0.0382 (9)	0.0041 (6)	0.0125 (6)	−0.0014 (7)
C5	0.0309 (7)	0.0280 (7)	0.0287 (7)	−0.0025 (6)	0.0109 (6)	−0.0038 (6)
C6	0.0269 (7)	0.0244 (7)	0.0233 (7)	−0.0005 (5)	0.0073 (5)	−0.0025 (5)
C7	0.0303 (7)	0.0246 (7)	0.0190 (6)	0.0010 (6)	0.0065 (5)	−0.0012 (5)
C8	0.0260 (6)	0.0226 (7)	0.0233 (7)	−0.0010 (5)	0.0059 (5)	−0.0021 (5)
C9	0.0312 (7)	0.0294 (7)	0.0261 (7)	0.0006 (6)	0.0010 (6)	−0.0025 (6)
C10	0.0304 (7)	0.0318 (8)	0.0345 (8)	0.0063 (6)	0.0028 (6)	−0.0016 (6)
C11	0.0327 (7)	0.0243 (7)	0.0277 (7)	0.0043 (6)	0.0072 (6)	−0.0017 (6)
C12	0.0323 (7)	0.0241 (7)	0.0236 (7)	0.0020 (6)	0.0088 (5)	−0.0016 (5)
C13	0.0245 (6)	0.0195 (6)	0.0232 (7)	−0.0018 (5)	0.0064 (5)	−0.0010 (5)
C14	0.0325 (7)	0.0267 (7)	0.0187 (6)	0.0019 (6)	0.0068 (5)	0.0003 (5)
C15	0.0492 (9)	0.0353 (8)	0.0269 (8)	−0.0090 (7)	0.0085 (6)	0.0001 (6)
C16	0.0987 (18)	0.0460 (12)	0.0520 (12)	−0.0294 (12)	0.0280 (12)	0.0052 (9)
C17	0.082 (2)	0.0543 (16)	0.070 (2)	−0.0088 (15)	0.0384 (17)	0.0188 (17)
C17A	0.082 (2)	0.0543 (16)	0.070 (2)	−0.0088 (15)	0.0384 (17)	0.0188 (17)
C18	0.0286 (7)	0.0242 (7)	0.0239 (7)	0.0051 (5)	−0.0003 (5)	−0.0014 (5)
C19	0.0338 (8)	0.0323 (8)	0.0256 (7)	0.0080 (6)	0.0015 (6)	−0.0029 (6)
C20	0.0479 (9)	0.0391 (9)	0.0326 (8)	0.0063 (7)	−0.0039 (7)	−0.0137 (7)
C21	0.0388 (9)	0.0307 (8)	0.0503 (10)	0.0005 (7)	−0.0097 (7)	−0.0093 (7)
C22	0.0344 (8)	0.0281 (8)	0.0434 (9)	0.0008 (6)	−0.0001 (7)	0.0019 (7)
C23	0.0328 (7)	0.0274 (7)	0.0280 (7)	0.0026 (6)	0.0021 (6)	−0.0002 (6)
C24	0.0588 (11)	0.0312 (9)	0.0514 (11)	0.0062 (8)	0.0094 (8)	−0.0059 (8)
C25	0.0405 (10)	0.0533 (11)	0.0463 (10)	0.0185 (8)	0.0037 (8)	−0.0019 (8)
C26	0.0469 (10)	0.0344 (8)	0.0418 (9)	0.0118 (7)	0.0124 (7)	−0.0053 (7)
C27	0.0409 (8)	0.0247 (7)	0.0363 (8)	−0.0019 (6)	0.0034 (6)	0.0005 (6)

### Geometric parameters (Å, º)

**Table:**

O1—C5	1.2426 (19)	C12—C13	1.5057 (18)
O2—C9	1.2185 (19)	C14—H14A	0.9900
O3—C15	1.217 (3)	C14—H14B	0.9900
O3A—C15	1.205 (11)	C14—C15	1.510 (2)
O4—C15	1.321 (2)	C16—H16A	0.9900
O4—C16	1.454 (2)	C16—H16B	0.9900
O5—H5	0.8400	C16—H16C	0.9900
O5—C19	1.364 (2)	C16—H16D	0.9900
N1—C1	1.3816 (18)	C16—C17	1.483 (4)
N1—C13	1.4091 (18)	C16—C17A	1.305 (9)
N1—C14	1.4633 (17)	C17—H17A	0.9800
C1—C2	1.504 (2)	C17—H17B	0.9800
C1—C6	1.365 (2)	C17—H17C	0.9800
C2—H2A	0.9900	C17A—H17D	0.9800
C2—H2B	0.9900	C17A—H17E	0.9800
C2—C3	1.533 (2)	C17A—H17F	0.9800
C3—C4	1.531 (2)	C18—C19	1.404 (2)
C3—C24	1.530 (2)	C18—C23	1.389 (2)
C3—C25	1.537 (2)	C19—C20	1.392 (2)
C4—H4A	0.9900	C20—H20	0.9500
C4—H4B	0.9900	C20—C21	1.381 (3)
C4—C5	1.504 (2)	C21—H21	0.9500
C5—C6	1.4401 (19)	C21—C22	1.377 (3)
C6—C7	1.514 (2)	C22—H22	0.9500
C7—H7	1.0000	C22—C23	1.390 (2)
C7—C8	1.5074 (18)	C23—H23	0.9500
C7—C18	1.5334 (19)	C24—H24A	0.9800
C8—C9	1.468 (2)	C24—H24B	0.9800
C8—C13	1.354 (2)	C24—H24C	0.9800
C9—C10	1.507 (2)	C25—H25A	0.9800
C10—H10A	0.9900	C25—H25B	0.9800
C10—H10B	0.9900	C25—H25C	0.9800
C10—C11	1.529 (2)	C26—H26A	0.9800
C11—C12	1.537 (2)	C26—H26B	0.9800
C11—C26	1.532 (2)	C26—H26C	0.9800
C11—C27	1.533 (2)	C27—H27A	0.9800
C12—H12A	0.9900	C27—H27B	0.9800
C12—H12B	0.9900	C27—H27C	0.9800
			
C15—O4—C16	117.16 (15)	O3—C15—O4	124.13 (18)
C19—O5—H5	109.5	O3—C15—C14	124.71 (17)
C1—N1—C13	119.22 (11)	O3A—C15—O4	120.8 (5)
C1—N1—C14	120.56 (12)	O3A—C15—C14	117.9 (5)
C13—N1—C14	120.23 (11)	O4—C15—C14	110.73 (13)
N1—C1—C2	117.03 (12)	O4—C16—H16A	110.0
C6—C1—N1	120.23 (13)	O4—C16—H16B	110.0
C6—C1—C2	122.68 (13)	O4—C16—H16C	108.4
C1—C2—H2A	108.7	O4—C16—H16D	108.4
C1—C2—H2B	108.7	O4—C16—C17	108.3 (2)
C1—C2—C3	114.28 (12)	H16A—C16—H16B	108.4
H2A—C2—H2B	107.6	H16C—C16—H16D	107.5
C3—C2—H2A	108.7	C17—C16—H16A	110.0
C3—C2—H2B	108.7	C17—C16—H16B	110.0
C2—C3—C25	108.42 (13)	C17A—C16—O4	115.5 (4)
C4—C3—C2	107.84 (13)	C17A—C16—H16C	108.4
C4—C3—C25	110.27 (14)	C17A—C16—H16D	108.4
C24—C3—C2	110.77 (14)	C16—C17—H17A	109.5
C24—C3—C4	110.08 (14)	C16—C17—H17B	109.5
C24—C3—C25	109.43 (15)	C16—C17—H17C	109.5
C3—C4—H4A	109.4	H17A—C17—H17B	109.5
C3—C4—H4B	109.4	H17A—C17—H17C	109.5
H4A—C4—H4B	108.0	H17B—C17—H17C	109.5
C5—C4—C3	111.22 (13)	C16—C17A—H17D	109.5
C5—C4—H4A	109.4	C16—C17A—H17E	109.5
C5—C4—H4B	109.4	C16—C17A—H17F	109.5
O1—C5—C4	119.99 (13)	H17D—C17A—H17E	109.5
O1—C5—C6	121.89 (14)	H17D—C17A—H17F	109.5
C6—C5—C4	118.08 (13)	H17E—C17A—H17F	109.5
C1—C6—C5	119.53 (13)	C19—C18—C7	121.14 (13)
C1—C6—C7	121.28 (12)	C23—C18—C7	121.01 (13)
C5—C6—C7	119.16 (12)	C23—C18—C19	117.85 (14)
C6—C7—H7	107.8	O5—C19—C18	122.76 (14)
C6—C7—C18	112.53 (11)	O5—C19—C20	116.89 (14)
C8—C7—C6	108.12 (11)	C20—C19—C18	120.34 (15)
C8—C7—H7	107.8	C19—C20—H20	119.8
C8—C7—C18	112.73 (11)	C21—C20—C19	120.35 (15)
C18—C7—H7	107.8	C21—C20—H20	119.8
C9—C8—C7	117.07 (12)	C20—C21—H21	119.9
C13—C8—C7	122.19 (13)	C22—C21—C20	120.17 (15)
C13—C8—C9	120.73 (13)	C22—C21—H21	119.9
O2—C9—C8	121.20 (14)	C21—C22—H22	120.2
O2—C9—C10	121.44 (14)	C21—C22—C23	119.56 (16)
C8—C9—C10	117.34 (13)	C23—C22—H22	120.2
C9—C10—H10A	109.3	C18—C23—C22	121.71 (14)
C9—C10—H10B	109.3	C18—C23—H23	119.1
C9—C10—C11	111.62 (12)	C22—C23—H23	119.1
H10A—C10—H10B	108.0	C3—C24—H24A	109.5
C11—C10—H10A	109.3	C3—C24—H24B	109.5
C11—C10—H10B	109.3	C3—C24—H24C	109.5
C10—C11—C12	107.90 (12)	H24A—C24—H24B	109.5
C10—C11—C26	110.38 (13)	H24A—C24—H24C	109.5
C10—C11—C27	109.47 (13)	H24B—C24—H24C	109.5
C26—C11—C12	109.08 (12)	C3—C25—H25A	109.5
C26—C11—C27	109.14 (13)	C3—C25—H25B	109.5
C27—C11—C12	110.87 (12)	C3—C25—H25C	109.5
C11—C12—H12A	108.9	H25A—C25—H25B	109.5
C11—C12—H12B	108.9	H25A—C25—H25C	109.5
H12A—C12—H12B	107.7	H25B—C25—H25C	109.5
C13—C12—C11	113.34 (12)	C11—C26—H26A	109.5
C13—C12—H12A	108.9	C11—C26—H26B	109.5
C13—C12—H12B	108.9	C11—C26—H26C	109.5
N1—C13—C12	117.39 (12)	H26A—C26—H26B	109.5
C8—C13—N1	120.15 (12)	H26A—C26—H26C	109.5
C8—C13—C12	122.36 (13)	H26B—C26—H26C	109.5
N1—C14—H14A	109.3	C11—C27—H27A	109.5
N1—C14—H14B	109.3	C11—C27—H27B	109.5
N1—C14—C15	111.74 (12)	C11—C27—H27C	109.5
H14A—C14—H14B	107.9	H27A—C27—H27B	109.5
C15—C14—H14A	109.3	H27A—C27—H27C	109.5
C15—C14—H14B	109.3	H27B—C27—H27C	109.5

### Hydrogen-bond geometry (Å, º)

**Table:**

*D*—H···*A*	*D*—H	H···*A*	*D*···*A*	*D*—H···*A*
O5—H5···O1	0.84	1.81	2.6319 (17)	166
C2—H2*A*···O2^i^	0.99	2.52	3.1663 (19)	123
C2—H2*B*···O5^i^	0.99	2.53	3.457 (2)	157
C12—H12*B*···O1^ii^	0.99	2.52	3.2708 (17)	133
C14—H14*A*···O1^ii^	0.99	2.53	3.3299 (18)	138
C14—H14*B*···O2^i^	0.99	2.66	3.473 (2)	140
C27—H27*B*···O3^iii^	0.98	2.51	3.452 (3)	161

Symmetry codes: (i) *x*+1/2, −*y*+1/2, *z*+1/2; (ii) *x*−1/2, −*y*+1/2, *z*+1/2; (iii) −*x*+1/2, *y*+1/2, −*z*+3/2.
